# Agricultural pest control with CRISPR‐based gene drive: time for public debate: Should we use gene drive for pest control?

**DOI:** 10.15252/embr.201744205

**Published:** 2017-05-16

**Authors:** Virginie Courtier‐Orgogozo, Baptiste Morizot, Christophe Boëte

**Affiliations:** ^1^ CNRS UMR7592Institut Jacques MonodUniversité Paris DiderotParisFrance; ^2^ CepercUMR 7304Aix‐en‐Provence Cedex 1France; ^3^ UMR “Emergence des Pathologies Virales”EPV Aix‐Marseille UniversitéIRD 190, INSERM 1207, EHESP, IHU Méditerranée InfectionMarseille Cedex 5France

**Keywords:** S&S: Economics & Business, S&S: Ecosystems & Environment, Synthetic Biology & Biotechnology

## Abstract

Gene drive technology to control disease vectors or pests has great potential for addressing humanitarian and public health problems. Its application for pest control in agriculture, however, raises important environmental, social and ethical issues.

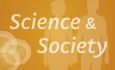

Gene drive based on the CRISPR/Cas‐9 gene editing system is a powerful technology that promotes the inheritance of the gene drive tool itself via sexual reproduction and can therefore spread quickly through a population. It holds great potential for public health and humanitarian purposes, such as reducing the burden of vector‐borne diseases like malaria. Here, we discuss another potential application of CRISPR‐based gene drive, namely the control of pest species to increase crop production. We argue that gene drive‐based pest control strategies should receive more attention from policymakers and the public given their enormous potential impact on the environment, their easy accessibility, and the current dearth of regulations.

## The CRISPR‐based gene drive technology

CRISPR‐based gene drive, named here gene drive for short, allows the rapid spread of a DNA cassette into a target species. The cassette contains three elements: a gene encoding the bacterial Cas‐9 protein, a gene coding a guide RNA that targets a particular site in the genome and flanking sequences which allow the cassette to insert at a given target site 1, 2, 3. This construct can copy and paste itself into a designed position within a genome, thereby propagating through a population. A normal allele has a 50% chance of being inherited by each offspring, but a gene drive cassette has more than 90% chance of being transmitted to the next generation owing to its ability to copy itself throughout the genome 1, 2, 3. Gene drive can bypass the vagaries of evolution, by manipulating both heredity and mutations: it enhances its transmission to the next generation, and mutations happen exactly where the gene drive has been designed to cut, producing the desired DNA sequence. In theory, the release of just a few individuals within a population could lead to complete invasion of the gene drive cassette within 15–20 generations 4.

## Wild GMOs

Any DNA sequence can be inserted into the gene drive cassette, which enables a wide range of applications: holding invasive species at bay, ensuring plants remain sensitive to herbicides, or rendering mosquitoes resistant to the malaria parasite 1. Gene drive technology is also perfectly suited for an additional aim that is not widely discussed in the media: the control of pest species to increase agricultural production. Compared to other pest management techniques, it is cheaper, more precise, and, so far, less controversial as, say, the use of pesticides. Gene drive‐mediated pest control can therefore be very attractive for agribusiness, because it allows direct manipulation of pest species, which is more complicated to achieve with classical GMO technologies; it may easily eradicate a species; and large effects are expected within just a few years after the release of the first gene drive organisms into the wild.

Classical techniques of genetic manipulation require an elaborate domestication relationship, including breeding of the target species for several generations, control of its reproduction, and interactions with humans. In contrast, gene drive technology can theoretically work in any species that reproduces sexually; it has already been successfully tested in yeasts, mosquitoes, flies, and human cells. Moreover, it just takes a few months and about US$1,000 worth of consumables to construct a gene drive organism. The major limitation is creating the first generation of individuals by injecting DNA and proteins into embryos or reproductive organs.

In theory, the release of just a few individuals within a population could lead to complete invasion of the gene drive cassette within 15–20 generations

If we define domestication as a series of operations—breeding, genetic modification, or gene editing—to manipulate and maintain a trait that is advantageous to humans, regardless of its effect on the domesticated individuals, then CRISPR‐based gene drive offers unprecedented power to domesticate almost any species. Agronomic science has been modifying *crop*s to increase productivity or resistance to pests or pathogens. Gene drive now allows manipulating *pests*. Organisms modified by gene drive can be considered as “wild GMOs”: this expression exemplifies the paradox that organisms can be gene‐edited and yet not engaged in any domestication relationship with us. Gene drive thus challenges the meaning of “natural” and “wild”.

## A fast‐growing technology

The gene drive cassette can be designed to transport a payload gene into the target genome to manipulate its physiology or metabolism. Alternatively, it can also be designed to integrate at any designated position within the genome to knock out a gene. If gene drive abolishes a female‐specific or male‐specific gene essential for reproduction, it can theoretically lead to extinction of the species 4. Since genes essential for reproduction or survival are easier to identify than genes that can finely alter species’ characteristics, eradicating a species appears to be more accessible than finely modifying its metabolism or physiology, especially in species for which we have only limited genetic knowledge.

Agronomic science has been modifying *crop*s to increase productivity or resistance to pests or pathogens. Gene drive now allows manipulating *pests*.

Research on gene drive and its applications is moving quickly. The CRISPR/Cas‐9 technology for gene editing is only 3–4 years old, and the first reports of gene drive organisms (laboratory fruit flies and mosquitoes) were published in 2015 1, 2, 3. While genetically modified organisms with a gene drive system have not yet been released, the US National Academy of Sciences, Engineering and Medicine recently approved research on gene drive and called for carefully controlled field trials 5. During the past 2 years, the Gates Foundation and the Indian Tata group invested more than US$140 million in gene drive research for controlling disease vectors and improving crop productivity. Bayer, Dupont, and Monsanto recently signed license agreements with biotech companies to use the CRISPR/Cas‐9 technology 6 (http://labiotech.eu/bayer-claims-crispr-patents-for-gene-editing-agreements/). Given the speed of progress, it may take only a few years until the first actors—companies, philanthropies, or research institutes—propose to release gene drive organisms into nature. It is therefore important to initiate debate about the implications of such releases, especially for pest control.

## Molecular precision and ecological unpredictability

The expression “gene editing” is often used to qualify the CRISPR technique and to underline its remarkable precision and efficiency at the molecular level. This conceals the absence of control at the ecosystem level where our knowledge remains speculative and patchy. Biologists who design gene drives are experts in molecular biology, without necessarily a deep understanding of community ecology or ecosystem dynamics. Yet, such ecological parameters are required to model the effects of gene drive on populations and ecosystems—demography and life history of the targeted population, interactions with other species and with other environmental elements, and so on. We therefore need a systemic scientific approach to move from uncertain risks to quantifiable hazards. This is a challenging endeavor and should be conducted with humility and patience.

In addition to the general risks associated with gene drive (Table 1), gene drive‐based pest control raises specific issues. A major concern is to determine *which species* is a *pest* for *which*. The malaria‐carrying mosquito is a pest for the human population on a big scale, and we are therefore compelled to consider all possible means to eradicate the disease, including gene drive. But the eventual success of such a strategy against human pests might become a Trojan horse to legitimate gene drive to control diverse pests without questioning to whom or to what they are harmful.

**Table 1:** Uncertain risks associated with gene drive in general

(I) **Escape**	Accidental laboratory release, cross‐breeding, or gene flow can potentially allow a drive to move beyond its target population(s)
(II) **Ride along**	It is possible that a foreign DNA sequence inserts itself within the gene drive cassette mid‐drive, thus allowing unwanted traits to “ride along” on the spreading drive. If this inserted DNA sequence brings up, unluckily, a benefit to its carriers and a detriment to humans, it will spread, for good and bad
(III) **Ecological impact**	Even when the new traits’ direct impact on a target population might be understood, the drive may have side effects on the surroundings, and these are difficult to estimate and quantify

“Reversal” drives have been proposed to undo the effects of a prior drive 9, but to our knowledge, all still leave a pseudo‐gene drive cassette within the genome.

The term “pest” is often used by agribusiness to designate species that diminish crop productivity. In this case, pests are not harmful to humans, but to specific economic interests—although, in times of food shortage, these different interests can converge. If manipulating other species to our benefit sounds at first like a humanitarian project, we should keep in mind that gene drive can also be used to serve the economic interests of particular groups with little concern for the general interest. There is no such thing as a “pest” *per se*: a population is only a pest with respect to specific interests, which does not mean these interests are illegitimate, only that they are *relative*. The species some call ‘pests’ may be the pollinators and the food of others species or may play an important ecological role for the local economy. Species are entangled in complex ecological interactions with other populations, which can be crucial to the functioning of local ecological dynamics.

## Adequacy between gene drive and agribusiness

In the future, gene drive could become a commonplace management technique for agribusiness, big or small, to edit the genome of the livings beings that hamper productivity. Given the lack of reliable modeling, it is safe to assume that normalizing the use of CRISPR‐based gene drive could lead to an ecological cacophony: every interest group in the agro‐food industry editing the genome of those they call pests, spreading various mutations through gene drive, and causing long‐term effects on the ecological dynamics of ecosystems—and on the human populations depending on them. We think such widespread use of gene drive is likely to happen, not because of a lack of ethics in agribusiness, but because of structural reasons: the adequacy between gene drive technique and the economic model of contemporary agribusiness regarding temporality and space.

The time‐frame of gene drive perfectly fits the economic development strategies dominant today in agribusiness, with a focus on short‐term return on investments and disdain for long‐term issues.

One of the main concerns over gene drive is its potential long‐term effects. The designated effects on the targeted populations will be fast—within a few years—while long‐term effects on ecosystems may take decades to appear and are extremely unpredictable. The time frame of gene drive perfectly fits the economic development strategies dominant today in agribusiness, with a focus on short‐term return on investments and disdain for long‐term issues. The current economical system based on productivity, yields, monoculture, and extractivism 7 is a perfect match for the operating mode of gene drive. In addition, agri‐food industry decision centers are rarely located near the production sites. They will be inclined to disregard the ecological long‐term risks as they only concern local human populations in their exploited lands. Gene drive then becomes an issue of environmental justice.

The scarce use of gene drive, if concerted, cautious and controlled, may not cause any ecological disaster, but the trivialization of the technique is clearly problematic. The spontaneous match between extractivist agriculture and gene drive could lead to multiple and uncoordinated releases of gene drives into the wild, which is likely to cause unpredictable ecological disturbances with far‐reaching consequences. At a time when an ecology consciousness is rising, recognizing the rights of wild species for survival and protection from careless human exploitation 8, the use of gene drive to fight pests should raise concerns.

To our knowledge, there is no regulation related to CRISPR‐based gene drive. Several groups of experts in France, Canada, and the USA have been examining various challenges raised by gene drive, but none have considered the widespread use of CRISPR‐based gene drive to eradicate pest species. Given the potential impact of this technology on society, it is time for a wider, public debate. An ethical debate on the right of humans to domesticate almost any species is needed. Lawmakers, stakeholders, and the civil society should join efforts to develop a dynamic governance of gene drive technology and its potential applications, especially for agricultural pest control.

## Conflict of interest

The authors declare that they have no conflict of interest.
